# Laparoscopic right paraduodenal hernia repair

**DOI:** 10.4103/0972-9941.68578

**Published:** 2010

**Authors:** James G Bittner

**Affiliations:** Department of Surgery, Medical College of Georgia School of Medicine, Augusta, Georgia, USA

Dear Sir,

I read with interest the recent case report submitted by Bhartia *et al*.,[1] which describes the laparoscopic repair of a symptomatic right paraduodenal hernia. After reviewing the article, the authors claimed their patient had the longest follow-up to date, stated that only three published reports have detailed laparoscopic right paraduodenal hernia repair, and claimed their patient’s postoperative hospital length of stay matched those of the patients in previous reports. Unfortunately, the authors did not include a time frame for follow-up but instead simply stated the patient “remains well till date and had no recurrence of abdominal symptoms.”[1] While their claim may be true, it is impossible for the reader to confirm this. At the time of case report submission, four published reports have been found to have detailed successful laparoscopic repair of right paraduodenal hernia. Perhaps the authors inadvertently overlooked a previously published article describing laparoscopic right paraduodenal hernia repair, complete with discussion and images of operative technique, embryology and literature review. Briefly, we reported the case of a 26-year-old female with symptoms suggestive of partial small-bowel obstruction and a 6-year history of intermittent abdominal pain. Plain abdominal radiographs and ultrasonography were non-diagnostic. Contrasted computed tomography of the abdomen revealed jejunum encased within the right upper quadrant, suspicious for right paraduodenal hernia. The patient underwent successful laparoscopic right paraduodenal repair and was discharged home on postoperative day 1 and is without sequelae 12 months postoperatively.[2]

With their report, the authors added to the growing number of reports of patients with right paraduodenal hernia managed safely and effectively using a laparoscopic technique and reminded the astute surgeon to expedite diagnosis and possibly shorten length of stay in the hospital by entertaining the diagnosis of paraduodenal hernia. This is particularly true in adults who present with acute symptoms associated with small-bowel obstruction. For surgeons with significant experience in minimally invasive techniques, physical examination and abdominal radiographs followed by diagnostic laparoscopy and definitive laparoscopic repair is a viable option for patients with acute and chronic presentation of right paraduodenal hernia.[2]

## References

[1] Bhartia V, Kumar A, Khedkar I, Savita KS, Goel N (2009). Laparoscopic repair of right paraduodenal hernia. J Minim Access Surg.

[2] Bittner JG, Edwards MA, Harrison SJ, Li K, Karmin PN, Mellinger JD (2009). Laparoscopic repair of a right paraduodenal hernia. JSLS.

